# Severe Diabetic Gastroparesis Masquerading as Emphysematous Gastritis and Surgical Abdomen

**DOI:** 10.7759/cureus.103510

**Published:** 2026-02-12

**Authors:** Mark Gamadia, Bianca Gamadia, Charles K Sonaliya

**Affiliations:** 1 Department of Medicine, Kansas City University, Kansas City, USA; 2 School of Osteopathic Medicine, Kansas City University, Kansas City, USA; 3 Department of Internal Medicine, Inspira Health, Millville, USA

**Keywords:** conservative medical management, gastric dysmotility, gastroparesis, portal venous gas, type 2 diabetes

## Abstract

A 66-year-old man with type 2 diabetes mellitus and known gastroparesis presented to the emergency department with fatigue, confusion, and hypoglycemia. During hospitalization, he developed worsening abdominal pain and distention. CT revealed marked gastric distention, extensive portal venous gas, and mesenteric air, raising concern for emphysematous gastritis, gastric outlet obstruction, or volvulus. Despite these alarming radiographic findings, the patient remained hemodynamically stable, with no peritoneal signs. Surgical consultation was obtained; however, given his clinical stability and history of gastroparesis, conservative management was pursued. Treatment included nasogastric decompression, IV fluids, and prokinetic therapy with metoclopramide and erythromycin. Within 48 hours, the patient demonstrated significant clinical and radiologic improvement, with resolution of portal venous gas and reduction in gastric distention. He was discharged on oral prokinetics and referred to a tertiary care center for ongoing gastroparesis management. This case highlights that severe diabetic gastroparesis can produce imaging findings that mimic life-threatening surgical emergencies. In hemodynamically stable patients with known motility disorders, recognition of this potential mimic is essential to avoid unnecessary surgical intervention and to guide appropriate conservative treatment.

## Introduction

Gastroparesis, most commonly seen in patients with long-standing diabetes mellitus, is defined as delayed gastric emptying in the absence of mechanical obstruction [1]. Symptoms can range from early satiety and bloating to more severe manifestations such as nausea, vomiting, and abdominal pain. In rare instances, gastric distention may become so pronounced that it produces radiographic findings suggestive of surgical emergencies, including gastric outlet obstruction, bowel ischemia, or emphysematous gastritis [2]. These overlapping clinical and imaging features, particularly portal venous gas, mesenteric air, and marked gastric distention, can create significant diagnostic uncertainty and often lead to urgent surgical consultations.

The pathogenesis of portal venous gas in the setting of severe gastroparesis is thought to involve mucosal disruption from progressive gastric overdistention, which allows translocation of intraluminal gas into the mesenteric and portal venous circulation [3]. While portal venous gas has historically been regarded as a harbinger of bowel necrosis carrying high mortality, an increasing number of benign etiologies have been recognized, including severe vomiting, gastric dilation, and functional dysmotility [2,4]. This distinction is clinically significant, as the decision to pursue surgical versus conservative management hinges on the integration of radiographic findings with clinical indicators. Current guidelines suggest that in hemodynamically stable patients without peritoneal signs, lactic acidosis, or pneumatosis with bowel wall thickening, conservative management is a reasonable initial approach, with close clinical and laboratory monitoring to detect early signs of deterioration [5].

We present a case of severe diabetic gastroparesis that mimicked a surgical abdomen, a phrase denoting an acute abdominal condition necessitating urgent or emergency surgical intervention, which was successfully managed with conservative treatment [6].

## Case presentation

A 66-year-old man with a medical history of type 2 diabetes mellitus, hypertension, hyperlipidemia, and previously diagnosed gastroparesis presented to the emergency department with fatigue and confusion. His initial blood glucose level was 72 mg/dL, which dropped to 40 mg/dL shortly after arrival. He was receiving intensive insulin therapy, including long-acting insulin degludec, 15 units twice daily, and rapid-acting insulin lispro, 9 units three times daily with meals. Admission laboratory findings were significant for hypoglycemia and an elevated HbA1c, reflecting poor long-term glycemic control, along with evidence of dehydration, but notably without lactic acidosis or significant leukocytosis (Table 1).

**Table 1:** Admission laboratory findings, including metabolic, hematologic, and renal parameters

Parameter	Patient value	Reference range
Glucose (initial)	72	70-100 mg/dL
Glucose (nadir)	40	70-100 mg/dL
White blood cell count	11.8	4.5-11.0 × 10⁹/L
Hemoglobin	14.2	13.5-17.5 g/dL
Platelet count	198	150-400 × 10⁹/L
Lactate	1.8	0.5-2.0 mmol/L
Sodium	132	136-145 mEq/L
Potassium	3.3	3.5-5.0 mEq/L
Bicarbonate	21	22-29 mEq/L
Blood urea nitrogen	28	7-20 mg/dL
Creatinine	1.4	0.7-1.3 mg/dL
Lipase	58	0-160 U/L
HbA1c	9.4	<5.7%

Laboratory evaluation revealed mild leukocytosis, likely secondary to physiologic stress, with lactate at the upper limit of normal. Notably, there was no lactic acidosis or bandemia to suggest bowel ischemia or necrotizing infection. Mild hyponatremia, hypokalemia, and prerenal azotemia were consistent with dehydration from prolonged poor oral intake.

During hospitalization, the patient developed worsening diffuse abdominal pain and visible distention. He was afebrile and hemodynamically stable but reported nausea and minimal oral intake. On physical examination, the abdomen was distended but nontender, without rebound or guarding.

**Figure 1:**
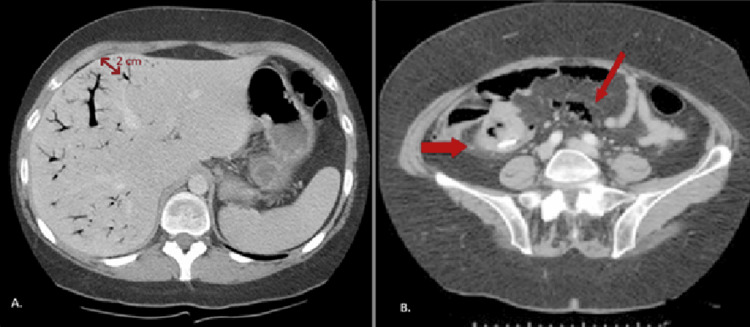
CT findings demonstrating portal venous gas and mesenteric air Initial CT of the abdomen and pelvis without contrast. (A) Axial image demonstrating extensive portal venous gas (arrows) with branching lucencies extending to within 2 cm of the liver capsule [3]. (B) Axial image showing a linear branching pattern of gas within the mesenteric veins (red arrow) with associated mild mesenteric stranding (red thick arrow) [7].

A CT of the abdomen and pelvis revealed marked gastric distention, extensive portal venous gas, and mesenteric air (Figure 1). The imaging was concerning for gastric outlet obstruction, emphysematous gastritis, or possible gastric volvulus. A surgical consultation was obtained because of concern for an emergent intra-abdominal process. However, the patient remained stable without signs of peritonitis. Given his known history of gastroparesis, the clinical team suspected that the imaging findings reflected gastric dysmotility rather than a true surgical emergency.

The patient was managed conservatively with nasogastric tube decompression, IV fluid resuscitation, and prokinetic therapy with metoclopramide and erythromycin. Over the next 48 hours, he demonstrated clinical improvement, and repeat imaging showed resolution of the portal venous gas with reduction in gastric distention. He was transitioned to a clear liquid diet and discharged with a referral to a tertiary care center for ongoing management of his gastroparesis.

Differential diagnosis

At presentation, the patient’s abdominal distention and radiographic findings raised concern for several life-threatening conditions. Initial CT revealed severe gastric dilation, extensive portal venous gas, and mesenteric air. These findings were highly suggestive of emphysematous gastritis, gastric outlet obstruction, or bowel ischemia, all of which warranted surgical consultation.

Emphysematous gastritis was considered due to the presence of portal venous gas and gastric wall abnormalities. However, the patient was afebrile, hemodynamically stable, and showed no systemic signs of infection or ischemia (Table 1), arguing against this diagnosis. Bowel ischemia was also a concern given the presence of mesenteric air. However, the absence of elevated lactate, a normal abdominal examination, and preserved vital signs made this less likely. Gastric outlet obstruction and volvulus were entertained because of the marked gastric distention, but imaging did not demonstrate a clear transition point or twisting of the gastric axis.

Ultimately, the patient’s history of diabetic gastroparesis, clinical stability, and favorable response to conservative management supported the diagnosis of severe gastroparesis with functional gastric outlet obstruction due to profound hypomotility, rather than a true surgical emergency (Figure 1).

Management and therapeutic approach

The patient was managed conservatively without surgical intervention. A nasogastric tube was inserted for gastric decompression, which immediately evacuated a large volume of retained gastric contents, estimated at over one liter, reflecting the severity of gastric distention. He maintained bowel rest and received IV fluid resuscitation to correct volume depletion and support perfusion. Electrolytes and glucose were closely monitored.

Pharmacologic treatment included prokinetic therapy with metoclopramide 10 mg intravenously three times daily and erythromycin 250 mg intravenously every six hours as a motilin receptor agonist. Both agents were selected to stimulate gastric motility and promote emptying. Insulin dosing was temporarily withheld during bowel rest to prevent further hypoglycemia and was resumed at reduced doses once oral intake was reinitiated. No antimicrobial therapy was initiated, as there were no signs of infection or systemic inflammation. The patient responded rapidly to this regimen, with resolution of abdominal symptoms and improvement on repeat imaging.

Clinical outcome and short-term follow-up

Within 48 hours of initiating conservative management, the patient experienced significant clinical improvement. His abdominal distention resolved, nausea decreased, and he was able to tolerate clear liquids. Repeat imaging showed resolution of portal venous gas and marked reduction in gastric distention.

He was discharged home on a prokinetic regimen of oral metoclopramide 10 mg three times daily before meals and instructed to follow a low-fat, low-fiber diet. Insulin dosing was adjusted to account for improved gastric motility and better oral intake. He was referred to a regional tertiary care center for multidisciplinary management of gastroparesis, including consideration of advanced therapies such as gastric electrical stimulation. At follow-up two weeks after discharge, he remained stable, with no recurrence of abdominal symptoms, improved glycemic control, and gradual return to daily activities.

## Discussion

Gastroparesis is a well-recognized complication of long-standing diabetes mellitus, resulting from autonomic neuropathy that impairs gastric motility [4]. While mild to moderate cases present with early satiety, bloating, or nausea, severe gastroparesis can lead to profound gastric distention. In rare cases, this can radiographically resemble critical intra-abdominal pathology, such as emphysematous gastritis, gastric outlet obstruction, or bowel ischemia [8,9].

Portal venous gas and mesenteric air are often considered alarming radiographic findings suggestive of bowel infarction or necrotizing gastrointestinal infection [6]. However, in this case, the absence of systemic toxicity, stable hemodynamics, and a reassuring abdominal examination shifted suspicion toward a functional, rather than surgical, etiology. This highlights the importance of integrating radiologic findings with clinical context.

The patient’s initial hypoglycemia may also be explained by insulin administration in the setting of delayed gastric emptying, leading to a mismatch between nutrient absorption and insulin action [1]. Management of severe gastroparesis involves bowel rest, decompression, and pharmacologic prokinetic agents. Metoclopramide and erythromycin remain commonly used first-line therapies [10].

Similar cases of severe gastroparesis mimicking a surgical abdomen have been reported in the literature, though they are uncommon. A few case reports describe gastric dilation and portal venous gas in patients with diabetes, initially misdiagnosed as bowel ischemia or emphysematous gastritis, and ultimately managed conservatively with good outcomes (Table 2) [2,4].

**Table 2:** Summary of published cases of gastroparesis mimicking surgical emergencies NGT, nasogastric tube

Study	Age/sex	Key imaging findings	Initial concern	Management	Outcome
Ball and Sharples (2013) [1]	62/M	Massive gastric dilation	Gastric outlet obstruction	Conservative: NGT decompression, prokinetics	Full recovery
Yeo and Dor (2025) [2]	55/M	Hepatic portal venous gas	Bowel ischemia	Conservative: observation, supportive care	Full recovery
Talluri et al. (2017) [4]	68/F	Portal venous gas, mesenteric venous gas, gastric dilation, bezoar	Bowel ischemia, emphysematous gastritis	Conservative: NGT decompression, IV fluids, prokinetics	Full recovery
Present case	66/M	Portal venous gas, mesenteric air, marked gastric distention	Emphysematous gastritis, gastric outlet obstruction, volvulus	Conservative: NGT decompression, IV fluids, metoclopramide, erythromycin	Full recovery at two-week follow-up

This case adds to the growing recognition that functional gastrointestinal disorders can present with imaging findings typically associated with surgical emergencies, including emphysematous gastritis, mesenteric ischemia, bowel perforation, and gastric volvulus. In this patient, emphysematous gastritis was excluded by the absence of fever, systemic toxicity, and gastric wall necrosis. Mesenteric ischemia was unlikely given normal lactate levels and the absence of peritoneal signs. Bowel perforation was not supported by the lack of free intraperitoneal air or clinical deterioration. Gastric volvulus was ruled out by the absence of a transition point or twisting of the gastric axis on imaging. Recognizing how each of these diagnoses was systematically excluded underscores the need for a cautious, multidisciplinary approach in diabetic patients with known motility disorders and concerning imaging.

## Conclusions

This case illustrates that severe diabetic gastroparesis may present with alarming imaging findings suggestive of surgical pathology, including portal venous gas, mesenteric air, and marked gastric distention. While these radiographic features have traditionally been associated with bowel ischemia and necrotizing infections carrying high mortality, they can also arise from benign causes such as profound gastric dysmotility. In hemodynamically stable patients with known motility disorders and no peritoneal signs, clinical context should guide decision-making before proceeding with surgical intervention. This case reinforces the value of a multidisciplinary approach and highlights the importance of referral to specialized centers for patients with refractory gastroparesis requiring advanced therapies.
